# Comparison of Readability of Official Public Health Information About COVID-19 on Websites of International Agencies and the Governments of 15 Countries

**DOI:** 10.1001/jamanetworkopen.2020.18033

**Published:** 2020-08-18

**Authors:** Vishala Mishra, Joseph P. Dexter

**Affiliations:** 1 Multidisciplinary Research Unit, Madras Medical College, Chennai, Tamil Nadu, India; 2 Neukom Institute for Computational Science, Dartmouth College, Hanover, New Hampshire

## Abstract

This cross-sectional study evaluates the readability of information on COVID-19 on websites of international agencies, governments of 15 countries, and US health departments.

## Introduction

Containment strategies for the coronavirus disease 2019 (COVID-19) pandemic have required broad public compliance, yet complex, contradictory, and false information proliferates.^1^ The American Medical Association (AMA), National Institutes of Health (NIH), and Centers for Disease Control and Prevention (CDC) recommend that medical information for the public be written at no higher than an eighth-grade reading level.^2^ We evaluated the readability of online information about COVID-19 provided by government and public health agencies and departments.

## Methods

For this cross-sectional study, between April 1, 2020, and April 5, 2020, we reviewed 18 websites, including 3 public health agency sites and 15 official government sites of countries with 5000 or more confirmed cases as of April 5 and with guidelines written in English. We identified pages about COVID-19 intended for a general audience, such as lists of frequently asked questions and fact sheets, and extracted the content into text files. This study followed the Strengthening the Reporting of Observational Studies in Epidemiology (STROBE) reporting guideline.

Readability grade levels based on 5 formulas (Flesch-Kincaid grade level [FKGL]; Simple Measure of Gobbledygook; Gunning Fog Index; Ford, Caylor, Sticht formula; and Coleman-Liau Index) were calculated using Readability Studio Professional, version 2019.3 (Oleander Software). Measures of syntactic complexity, including mean length of clause and dependent clauses per T-unit, were computed using the L2 Syntactic Complexity Analyzer, version 3.3.3.^3^

We evaluated web pages against grade level recommendations of the AMA, NIH, and CDC; the CDC pages were evaluated using their health literacy guidelines (a reading level of grade 8, 1 to 2 syllables per word, 8 to 10 words per sentence, and substitution of “everyday” synonyms for 121 difficult terms related to public health).^2,4,5^ Sample passages from the websites with varying FKGL are provided in the eAppendix in the Supplement. State literacy data were obtained from a previous survey.^6^

Statistical calculations were performed using Stata, version 13 (StataCorp). A Wilcoxon rank-sum test was used for 2-sample comparisons, and correlation was assessed by Spearman *r*. Statistical significance was defined as *P* < .05.

## Results

Among all 18 websites evaluated, information about COVID-19 uniformly exceeded the recommended reading level of grades 6 through 8 (Table). All pages (n = 149) drawn from the websites scored above 8.0 by at least 1 metric, and 141 (95%) scored above 8.0 by all 5 metrics. A total of 145 pages (97%) exceeded the syntactic complexity typical for text written at a grade 8 level (mean length of clause, 8.0; dependent clauses per T-unit, 0.2).

**Table.** Readability of COVID-19 Information From the WHO, CDC, ECDC, and Governments of 15 Countries

Resource	Web pages, No.	Readability formulas, median (IQR) [range]					Syntactic complexity, median (IQR) [range]^a^	
		FKGL	SMOG	GFI	FORCAST	CLI	MLC	DC/T
WHO	12	11.8 (4.0) [8.7-16.7]	13.6 (3.6) [11.2-17.3]	13.6 (4.7) [10.1-15.6]	11.5 (1.3) [10.4-12.4]	12.4 (3.2) [10.5-16.2]	12.5 (2.6) [10.6-16.3]	0.5 (0.4) [0.3-0.9]
CDC	68	11.0 (3.2) [5.4-16.6]	13.4 (2.5) [8.1-17.4]	12.7 (3.0) [6.8-18.4]	11.4 (0.9) [8.7-13.4]	12.8 (2.9) [8.1-18.6]	11.6 (2.0) [7.4-15.1]	0.5 (0.2) [0.2-2.5]
ECDC	1	13.1	15.0	14.0	11.6	12.9	12.5	0.5
US^b^	19	11.5 (1.9) [8.8-14.9]	13.5 (1.4) [11.6-15.7]	12.4 (2.1) [10.7-14.9]	11.3 (0.7) [10.6-12.1]	12.2 (1.8) [10.9-15.2]	11.8 (2.6) [10.1-14.1]	0.6 (0.3) [0.3-0.8]
Australia	14	10.7 (1.6) [8.6-11.7]	12.8 (1.6) [11.2-14.0]	11.8 (1.8) [9.9-13.7]	10.7 (0.5) [9.8-11.4]	11.2 (1.7) [9.5-14.1]	10.9 (1.4) [9.4-15.8]	0.6 (0.3) [0.4-0.8]
Austria	1	12.0	14.1	12.4	11.2	11.9	12.3	0.4
Belgium	1	9.4	11.9	11.3	11.3	11.6	10.3	0.3
Canada	1	10.5	13.1	11.5	11	11.5	10.2	0.6
France	1	9.4	11.9	9.9	10.2	10.9	11.1	0.6
Germany	1	11.7	13.9	12.2	11.2	12.3	12.2	0.4
Israel	1	11.3	13.2	12.4	11.1	11.5	12.5	0.4
Italy	1	12.6	14.2	12.6	11.3	12.5	14.4	0.4
Netherlands	2	7.8 (0.7) [7.4-8.1]	10.2 (0.9) [9.7-10.6]	8.7 (0.9) [8.2-9.1]	10.3 (0) [10.3-10.3]	9.2 (0.6) [8.9-9.5]	9.4 (0.2) [9.3-9.5]	0.5 (0) [0.5-0.5]
Norway	13	11.1 (3.2) [7.0-13.9]	12.3 (1.9) [10.6-15.2]	12.1 (1.9) [10.0-14.5]	10.8 (1.3) [9.8-12.5]	11.4 (3.1) [8.7-15.3]	9.9 (1.9) [7.4-13.9]	0.7 (0.2) [0.4-1.2]
South Korea	1	11.5	13.4	12.4	11.4	12.9	11.6	0.5
Sweden	1	10.6	12.7	11.8	11.1	11.5	10.1	0.6
Switzerland	1	9.2	11.9	10.7	10.6	10.8	10.2	0.5
UK^c^	10	11.6 (2.4) [8.4-15.1]	12.9 (1.9) [11.2-16.1]	12.8 (3.2) [9.7-15.2]	10.3 (1.0) [9.6-11.6]	10.8 (1.7) [8.7-13.7]	10.4 (1.1) [9.3-15.0]	0.8 (0.4) [0.5-1.2]

Abbreviations: CDC, Centers for Disease Control and Prevention; CLI, Coleman-Liau Index; COVID-19, coronavirus disease 2019; DC/T, dependent clauses per T-unit; ECDC, European Centre for Disease Control and Prevention; FKGL, Flesch-Kincaid grade level; FORCAST, Ford, Caylor, Sticht formula; GFI, Gunning Fog Index; MLC, mean length of clause; SMOG, Simple Measure of Gobbledygook; WHO, World Health Organization.

^a^ The MLC measures elaboration at the clause level (number of words per clause), and DC/T measures clausal subordination.

^b^ Official guidelines provided by the White House Coronavirus Task Force were included.

^c^ Official guidelines provided by Public Health England were included.

Across all CDC pages, the median FKGL was 11.0 (interquartile range [IQR], 3.2; range, 5.4-16.6). Median syllables per word was 1.7 (IQR, 0.2; range, 1.3-2.0), median words per sentence was 15.6 (IQR, 3.2; range, 8.2-31.5), and 67 pages (99%) used at least 1 difficult term. Median number of difficult terms used was 11.0 (IQR, 10.5; range, 0-50).

The FKGL was above 8.0 for every state (Figure). Compared with the CDC, states (median, 24.5; IQR, 19.0; range, 4.0-53.0) used significantly more difficult terms (*P* < .001). Use of difficult terms by states was correlated with FKGL (Spearman *r* = 0.36; 95% CI, 0.09-0.58; *P* = .01). Nine of the 10 states with the highest illiteracy rates had information written above a grade 10 level.^6^

**Figure.**
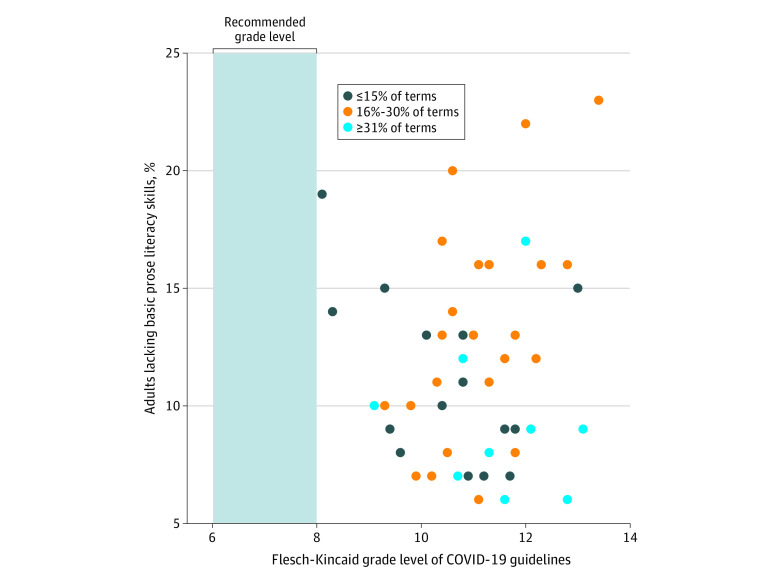
Readability, Literacy, and Use of Difficult Terms Across All 50 US States For each state, an official website with frequently asked questions or related information for the public was assessed for readability and use of the 121 difficult words and phrases discussed in the Centers for Disease Control and Prevention’s health literacy guidelines.^5^ Because 3 pairs of states that used 16% to 30% of the difficult terms had identical Flesch-Kincaid grade levels and literacy, only 47 data points are shown. COVID-19 indicates coronavirus disease 2019.

## Discussion

We found that official information about COVID-19 exceeded the recommended reading level, exhibited complex syntax, and used technical terminology. The significant difference in use of difficult terms between the CDC and state resources may reflect the influence of federal oversight mandating government communication that is understandable to the public. Limitations included the focus on text, with no evaluation of multimedia communication, and lack of data about actual comprehension or relevant outcomes such as adherence to mitigation strategies.

Nonadherence to readability standards may have a greater influence in communities with lower health literacy, potentially exacerbating the disparate effects of the pandemic. As such, efforts should focus on the urgent development of plain-language COVID-19 resources that conform to established guidelines for clear communication and are more accessible to all audiences.

## Notes

Supplement. **eAppendix.** Sample Passages About COVID-19 Written at Different Flesch-Kincaid Grade Levels
