# Supplementary material for: Comparison of Readability of Official Public Health Information About COVID-19 on Websites of International Agencies and the Governments of 15 Countries
Source: JAMA Netw Open. 2020 Aug 18;3(8):e2018033. doi: 10.1001/jamanetworkopen.2020.18033 (PMC7435342; doi:10.1001/jamanetworkopen.2020.18033)
Supplement: Supplement. — eAppendix. Sample Passages About COVID-19 Written at Different Flesch-Kincaid Grade Levels [file jamanetwopen-3-e2018033-s001.pdf]

Supplementary Online Content

Mishra V, Dexter JP. Comparison of readability of official public health information about COVID-19 on websites of international agencies and the governments of 15 countries. *JAMA Netw Open*. 2020;3(8):e2018033. doi:10.1001/jamanetworkopen.2020.18033

eAppendix. Sample Passages About COVID-19 Written at Different Flesch-Kincaid Grade Levels

This supplementary material has been provided by the authors to give readers additional information about their work.

© 2020 Mishra V et al. *JAMA Network Open*.

eAppendix. Sample Passages About COVID-19 Written at Different Flesch-Kincaid Grade Levels

This appendix provides six sample passages about COVID-19 written at Flesch-Kincaid grade levels (FKGL) ranging from 7.4 to 16.0. The passages are short excerpts from a selection of the websites reviewed in the study. The contents of each website may have changed since the study was conducted, and the formatting and spacing of text in the eAppendix may differ from its original online presentation.

The full corpus of passages used in the study is available at www.github.com/qcrit/COVID-19-readability.

Netherlands, "Frequently asked questions about coronavirus and health" (FKGL = 7.4)

https://www.government.nl/topics/coronavirus-covid-19/frequently-asked-questions-about-coronavirus-and-health

What are the symptoms of coronavirus disease?

Symptoms include fever in combination with coughing, shortness of breath or pneumonia.

I think I have coronavirus. What should I do?

For everyone in the Netherlands: stay at home if you have mild cold-like symptoms such as a runny nose, sneezing, a sore throat, a cough or a fever below 38 degrees Celsius. Avoid social contacts. This means, for instance, that you may not have any visitors or visit anyone else, and you may not do any shopping. Only call your doctor if the symptoms worsen and you require medical assistance. If you feel better and have not had any symptoms for 24 hours, you have recovered. You can no longer infect others.

What can I do to prevent coronavirus from spreading?

Stay home as much as possible. You should work from home if you can. You should only go outside to shop for essentials, to get some fresh air or to care for another person. Always stay 1.5 metres from other people and avoid gathering in groups. If you have a cough and/or cold, stay at home. If you or someone in your household develops a fever or shortness of breath, all the members of the household must stay at home. Wash your hands regularly with water and soap. Cough and sneeze into the crook of your elbow. Use paper tissues and discard them after use. Don't shake hands with others. Stay at least 1.5 metres away from other people.

How do I know if someone in my vicinity has been infected with coronavirus?

This is published by your municipal health service.

How many people in the Netherlands have contracted coronavirus?

See the counter with the number of infections in the Netherlands on the RIVM website.

I live with someone who has a fever and shortness of breath. What should I do?

All the members of the household should stay home, even if they have no symptoms. If it is necessary to do grocery shopping, this should be done by a member of the household who has no symptoms. You may not have any visitors.

© 2020 Mishra V et al. *JAMA Network Open*.

Only call your doctor if the symptoms worsen or if you require medical assistance. All members of the household may leave the house again once no one has had any symptoms for 24 hours. Make sure you practise good hygiene.

What are mild cold-like symptoms?

Mild cold-like symptoms include: a fever below 38°C.

How far away does ‘keeping your distance’ actually mean?

Keeping your distance means staying at least 1.5 metres away from others, to protect yourself and others from a coronavirus infection. Coughing and sneezing spread tiny droplets through the air that contain the virus. People who breathe in these droplets can be infected. When you sneeze, these droplets rarely go further than 1.5 metres. If you stay 1.5 metres away from others, you are still allowed to do the shopping, go for a walk, ride your bicycle and pick up takeaway food.

Is there a self-test that I can use to check if I’m infected with coronavirus?

No, there are no approved coronavirus self-test kits on the market at this time. Any self-tests developed must first be assessed by a notified body that is registered with the Health and Youth Care Inspectorate. Currently, no self-tests for coronavirus have been approved.

Can I pick up new coronavirus by touching a contaminated surface or object like a door handle, ATM, handrail or money?

A virus always needs a person or animal to ‘stay alive’ and multiply. Coronavirus spreads through people. Outside the body the virus only stays active for a short while, depending on the type of surface, the temperature and humidity level. Viruses usually survive for longer periods on hard, smooth materials like plastics, metal and glass than on porous materials like paper and cardboard. The chance of getting coronavirus from touching a surface or object is very small. Make sure you take measures that you would normally take to protect yourself against viruses that cause flu and common colds, like washing your hands regularly.

Do I run a greater risk of coronavirus infection if I smoke?

Not much research has been done yet into smoking and coronavirus. Anyone can be infected. If you smoke, however, the illness may take a more severe form.

Norway, “The new coronavirus (COVID-19) ” (FKGL = 9.2)

https://www.fhi.no/contentassets/37298bcaf6724377b68018cfd7db4309/vedlegg/english_generell-informasjon-korona.pdf

The new coronavirus is attracting a lot of attention. Most people who develop coronavirus disease (COVID-19) only suffer mild symptoms, but some people can become very ill. This is why we are trying to slow the spread of infection across the country.

Some people have been asked by their doctor to stay at home. Anyone who is diagnosed with COVID-19 must be completely isolated from other people through so-called ‘home isolation’. Anyone who has not been diagnosed with COVID-19, but may have been exposed to infection, must be placed in so-called ‘home quarantine’.

© 2020 Mishra V et al. *JAMA Network Open*.

What does ‘home isolation’ mean?

If your doctor has told you that you have COVID-19 or if you are waiting for your test results, you must avoid all contact with other people so that you do not infect others.

Home isolation means that you must be isolated from everyone else, including people you live with. You must not go out, you should remain in a room by yourself, eat in the room and not share towels with anyone else.

If your condition deteriorates, contact your doctor by phone. The doctor may also decide that you need to be isolated in hospital. Isolation lasts until your doctor tells you that you are infection-free or until your test result shows that you do not have COVID-19.

What does ‘home quarantine’ mean?

If your doctor has told you that you are not ill, but that you have been exposed to infection, you must go into quarantine. You must stay at home, but home quarantine is not as strict as home isolation.

Home quarantine means you must reduce your contact with other people as much as possible, but you do not have to be completely isolated. You must not go to school or work, or do any other activities, and you must not travel on public transport (bus, tram, underground, train, air or ferry), but you can go for walks on your own.

If you start coughing, develop a throat infection or fever or experience difficulty breathing, contact a doctor by phone. Home quarantine lasts for 14 days after you have been exposed to infection.

World Health Organization, “Myth busters” (FKGL = 10.8)

https://www.who.int/emergencies/diseases/novel-coronavirus-2019/advice-for-public/myth-busters

From the evidence so far, the COVID-19 virus can be transmitted in ALL AREAS, including areas with hot and humid weather. Regardless of climate, adopt protective measures if you live in, or travel to an area reporting COVID-19. The best way to protect yourself against COVID-19 is by frequently cleaning your hands. By doing this you eliminate viruses that may be on your hands and avoid infection that could occur by then touching your eyes, mouth, and nose.

Cold weather and snow CANNOT kill the new coronavirus. There is no reason to believe that cold weather can kill the new coronavirus or other diseases. The normal human body temperature remains around 36.5°C to 37°C, regardless of the external temperature or weather. The most effective way to protect yourself against the new coronavirus is by frequently cleaning your hands with alcohol-based hand rub or washing them with soap and water.

Taking a hot bath will not prevent you from catching COVID-19. Your normal body temperature remains around 36.5°C to 37°C, regardless of the temperature of your bath or shower. Actually, taking a hot bath with extremely hot water can be harmful, as it can burn you. The best way to protect yourself against COVID-19 is by frequently cleaning your hands. By doing this you eliminate viruses that may be on your hands and avoid infection that could occur by then touching your eyes, mouth, and nose.

The new coronavirus CANNOT be transmitted through mosquito bites. To date there has been no information nor evidence to suggest that the new coronavirus could be transmitted by mosquitoes. The new coronavirus is a respiratory virus which spreads primarily through droplets generated when an infected person coughs or sneezes, or through droplets of saliva or discharge from the nose. To protect yourself, clean your hands frequently with an

© 2020 Mishra V et al. *JAMA Network Open*.

alcohol-based hand rub or wash them with soap and water. Also, avoid close contact with anyone who is coughing and sneezing.

Are hand dryers effective in killing the new coronavirus?

No. Hand dryers are not effective in killing the 2019-nCoV. To protect yourself against the new coronavirus, you should frequently clean your hands with an alcohol-based hand rub or wash them with soap and water. Once your hands are cleaned, you should dry them thoroughly by using paper towels or a warm air dryer.

Can an ultraviolet disinfection lamp kill the new coronavirus?

UV lamps should not be used to sterilize hands or other areas of skin as UV radiation can cause skin irritation.

How effective are thermal scanners in detecting people infected with the new coronavirus?

Thermal scanners are effective in detecting people who have developed a fever (i.e have a higher than normal body temperature) because of infection with the new coronavirus. However, they cannot detect people who are infected but are not yet sick with fever. This is because it takes between 2 and 10 days before people who are infected become sick and develop a fever.

Canada, "Coronavirus disease (COVID-19)" (FKGL = 11.5)

https://www.canada.ca/en/public-health/services/diseases/coronavirus-disease-covid-19.html

Should the general population in Canada wear masks to protect themselves from COVID-19?

Medical masks, including surgical, medical procedure face masks and respirators (like N95 masks), must be kept for health care workers and others providing direct care to COVID-19 patients.

Wearing a non-medical mask or face covering while out in public is recommended for periods of time when it is not possible to consistently maintain a 2-metre physical distance from others, particularly in crowded public settings, such as:

- stores
- shopping areas
- public transportation

Public health officials will make recommendations based on a number of factors, including the rates of infection and/or transmission in the community. Recommendations may vary from location to location.

If you do choose to wear one, refer to the:

- guidelines on wearing non-medical masks and how to make your own
- COVID-19 Special Advisory Committee's recommendations on the use of non-medical cloth masks or face coverings in community settings

Masks alone will not prevent the spread of COVID-19. You must consistently and strictly adhere to good hygiene and public health measures, including frequent hand washing and physical (social) distancing.

© 2020 Mishra V et al. *JAMA Network Open*.

Can COVID-19 be transmitted through food?

There is currently no evidence to suggest that food is a likely source or route of transmission of the virus and there are currently no reported cases of COVID-19 transmission through food. People are unlikely to be infected with the virus through food. Scientists and food safety authorities across the world are closely monitoring the spread of COVID-19. If we become aware of a potential food safety risk, appropriate actions will be taken to ensure the safety of Canada's food supply. Coronaviruses are killed by common cleaning and disinfection methods and by cooking food to safe internal temperatures. Learn more about food safety and healthy eating during the COVID-19 pandemic.

Is there a vaccine or therapy that can treat or prevent COVID-19?

At this time, a vaccine and therapies to treat or prevent this disease have not yet been identified. But research and development are underway. The COVID-19 pandemic has resulted in a global review of therapies that may be used to treat or prevent the disease. Health Canada is fast tracking the importation and sale of medical devices for use in relation to COVID-19. If you have received a flu vaccine, it will not protect against coronaviruses, but will help prevent the flu. Getting the flu could make you more vulnerable to other infections. Getting the flu vaccine will not increase your risk of illness from coronavirus. For more information, please refer to this recently published Canadian research study.

United States of America, "Find answers about Coronavirus" (FKGL = 13.8)

https://faq.coronavirus.gov/

Why might someone blame or avoid individuals and groups (create stigma) because of COVID-19?

People in the U.S. may be worried or anxious about friends and relatives who are living in or visiting areas where COVID-19 is spreading. Some people are worried about getting the disease from these people. Fear and anxiety can lead to social stigma, for example, toward people who live in certain parts of the world, people who have traveled internationally, people who were in quarantine, or healthcare professionals.

Stigma is discrimination against an identifiable group of people, a place, or a nation. Stigma is associated with a lack of knowledge about how COVID-19 spreads, a need to blame someone, fears about disease and death, and gossip that spreads rumors and myths.

Stigma hurts everyone by creating more fear or anger toward ordinary people instead of focusing on the disease that is causing the problem.

What is a novel coronavirus?

A novel coronavirus is a new coronavirus that has not been previously identified. The virus causing coronavirus disease 2019 (COVID-19), is not the same as the coronaviruses that commonly circulate among humans and cause mild illness, like the common cold.

Coronavirus disease 2019 (COVID-19) is a respiratory illness that can spread from person to person. There are many types of human coronaviruses, including some that commonly cause mild upper-respiratory tract illnesses. COVID-19 is a new disease, caused by a novel (or new) coronavirus that has not previously been seen in humans.

Read about COVID-19 Symptoms

© 2020 Mishra V et al. *JAMA Network Open*.

Why is the disease being called coronavirus disease 2019, COVID-19?

On February 11, 2020 the World Health Organization announced an official name for the disease that is causing the 2019 novel coronavirus outbreak, first identified in Wuhan China. The new name of this disease is coronavirus disease 2019, abbreviated as COVID-19. In COVID-19, 'CO' stands for 'corona,' 'VI' for 'virus,' and 'D' for disease. Formerly, this disease was referred to as "2019 novel coronavirus" or "2019-nCoV".

There are many types of human coronaviruses including some that commonly cause mild upper-respiratory tract illnesses. COVID-19 is a new disease, caused be a novel (or new) coronavirus that has not previously been seen in humans.

World Health Organization, "Q&A: HIV, antiretrovirals and COVID-19" (FKGL = 15.1)

https://www.who.int/emergencies/diseases/novel-coronavirus-2019/question-and-answers-hub/q-a-detail/q-a-on-covid-19-hiv-and-antiretrovirals

Are people living with HIV at increased risk of being infected with the virus that causes COVID-19?

People living with HIV with advanced disease, those with low CD4 and high viral load and those who are not taking antiretroviral treatment have an increased risk of infections and related complications in general. It is unknown if the immunosuppression of HIV will put a person at greater risk for COVID-19, thus, until more is known, additional precautions for all people with advanced HIV or poorly controlled HIV, should be employed [1],[2].

At present there is no evidence that the risk of infection or complications of COVID-19 is different among people living with HIV who are clinically and immunologically stable on antiretroviral treatment when compared with the general population. Some people living with HIV may have known risk factors for COVID-19 complications, such as diabetes, hypertension and other noncommunicable diseases and as such may have increased risk of COVID-19 unrelated to HIV. We know that during the SARS and MERS outbreaks there were only a few case reports of mild disease among people living with HIV.

To date, there is a case report of a person living with HIV who had COVID-19 and recovered [3] and a small study on risk factors and antiretrovirals used among people living with HIV with COVID-19 from China. This study reported similar rates of COVID-19 disease as compared to the entire population and increased risk with older age, but not with low CD4, high viral load level or antiretroviral regimen [4]. Current clinical data suggest the main mortality risk factors are linked to older age and other comorbidities including cardiovascular disease, diabetes, chronic respiratory disease, and hypertension. Some very healthy people have also developed severe disease from the coronavirus infection [5].

PLHIV are advised to take the same precautions as the general population [6],[7]:

- wash hands often
- cough etiquette
- physical distancing
- seek medical care if symptomatic
- self-isolation if in contact with someone with COVID-19 and other actions per the government response
- People living with HIV who are taking antiretroviral drugs should ensure that they have at least 30 days and up to 6-month supply of medicines and ensure that their vaccinations are up to date (influenza and

© 2020 Mishra V et al. *JAMA Network Open*.

pneumococcal vaccines). Adequate supplies of medicines to treat co-infections and comorbidities and addiction should also be ensured.

Can antiretrovirals be used to treat COVID-19?

Several studies have suggested that patients infected with the virus causing COVID-19, and the related coronavirus infections (SARS-CoV and MERS-CoV) had good clinical outcomes, with almost all cases recovering fully. In some cases, patients were given an antiretroviral drug: lopinavir boosted with ritonavir (LPV/r). These studies were mostly carried out in HIV-negative individuals. It is important to note that these studies using LPV/r had important limitations. The studies were small, timing, duration and dosing for treatment were varied and most patients received co-interventions/co-treatments which may have contributed to the reported outcomes. While the evidence of benefit of using antiretrovirals to treat coronavirus infections is of very low certainty, serious side effects were rare. Among people living with HIV, the routine use of LPV/r as treatment for HIV is associated with several side effects of moderate severity. However, as the duration of treatment in patients with coronavirus infections was generally limited to a few weeks, these occurrences can be expected to be low or less than that reported from routine use.

© 2020 Mishra V et al. *JAMA Network Open*.
